# Endo-microscopy beyond the Abbe and Nyquist limits

**DOI:** 10.1038/s41377-020-0308-x

**Published:** 2020-05-07

**Authors:** Lyubov V. Amitonova, Johannes F. de Boer

**Affiliations:** 1 LaserLaB, Department of Physics and Astronomy, Vrije Universiteit Amsterdam, De Boelelaan 1081, 1081 HV Amsterdam, The Netherlands; 2 Advanced Research Center for Nanolithography (ARCNL), Science Park 106, 1098 XG Amsterdam, The Netherlands

**Keywords:** Super-resolution microscopy, Imaging and sensing, Fibre optics and optical communications

## Abstract

For several centuries, far-field optical microscopy has remained a key instrument in many scientific disciplines, including physical, chemical, and biomedical research. Nonetheless, far-field imaging has many limitations: the spatial resolution is controlled by the diffraction of light, and the imaging speed follows the Nyquist–Shannon sampling theorem. The recent development of super-resolution techniques has pushed the limits of spatial resolution. However, these methods typically require complicated setups and long acquisition times and are still not applicable to deep-tissue bioimaging. Here, we report imaging through an ultra-thin fibre probe with a spatial resolution beyond the Abbe limit and a temporal resolution beyond the Nyquist limit simultaneously in a simple and compact setup. We use the random nature of mode coupling in a multimode fibre, the sparsity constraint and compressive sensing reconstruction. The new approach of super-resolution endo-microscopy does not use any specific properties of the fluorescent label, such as depletion or stochastic activation of the molecular fluorescent state, and therefore can be used for label-free imaging. We demonstrate a spatial resolution more than 2 times better than the diffraction limit and an imaging speed 20 times faster than the Nyquist limit. The proposed approach can significantly expand the realm of the application of nanoscopy for bioimaging.

## Microscopy: Bio-imaging beyond limits

A new procedure for imaging living cells and tissues using an ultra-fine optical fibre to detect light from fluorescent dyes overcomes fundamental limits restricting existing methods. Lyubov Amitonova and Johannes de Boer developed and demonstrated the technology at the Free University of Amsterdam in the Netherlands. Optical imaging has previously been subject to constraints known as the Abbe and Nyquist limits. The Abbe limit sets the smallest distance that can be resolved at about half the wavelength of the light. The Nyquist limit determines the fastest rate at which a light signal can be sampled. The researchers have achieved fluorescence microscopy more than three times better than the Abbe limit and 20 times faster than the Nyquist limit. Their breakthrough is based on innovative light manipulation and sampling procedures in a simple and compact set-up.

## Introduction

Optical techniques have long been recognized as indispensable tools for bioimaging. Modern microscopy demonstrates a drive towards miniaturization caused by the need to access deep tissues *in vivo*^1^. Miniaturized endo-microscopy provides large depth penetration and is not limited by the interior of a hollow organ or cavity of the body. The recent emergence of spatial wavefront shaping^2,3^ has allowed a conventional step-index multimode (MM) fibre to be utilized as an ultra-thin aberration-free imaging probe^4–7^. The numerical aperture (NA) of MM fibres approaches 0.9^8,9^, paving the way towards high-resolution but still diffraction-limited imaging. Currently, minimally invasive endoscopes are widely used in neuroscience for *in vivo* deep brain imaging^10–12^, as well as in clinical studies to assist in detecting cancers, to prescribe the right drugs and to monitor treatment response^13,14^. The ongoing challenge towards high-quality minimally invasive deep-tissue imaging calls for a new solution that combines enhancement in the spatial and temporal resolutions with a footprint reduction.

Recent years have witnessed the development of super-resolution far-field fluorescence microscopy that allows the diffraction-limited resolution to be surpassed, unveiling processes at the nano-scale level^15^. Structured illumination microscopy utilizes spatial modulation of the fluorescence emission with patterned illumination^16^. However, it yields a resolution improved by only a factor of two. Stochastic optical reconstruction microscopy and photoactivation localization microscopy (PALM) are based on the stochastic switching on of individual molecules at different times^17,18^. Stimulated emission depletion (STED) microscopy increases the resolution through shrinking the point-spread function (PSF) by depleting the fluorescence emission in the periphery of the diffraction-limited spot^19^. These techniques have specific requirements for fluorescent labels.

Parallel efforts aim to improve the imaging speed^20^. An imaging approach with a millisecond time resolution is required to study fast processes, such as action potentials and communication in neuronal networks. Not surprisingly, advanced methods of diffraction-unlimited resolution are time-consuming, and the trade-off between the spatial and the temporal resolution affects all super-resolution techniques. Most approaches require point-by-point scanning, and consequently, the acquisition speed is limited by the Nyquist–Shannon sampling theorem^21^.

Achieving super-resolution *in vivo* deep in living tissue is extremely challenging due to the limited optical access, optical aberrations, and low imaging speed. The maximum demonstrated imaging depth for super-resolution is still only 120 μm below the tissue surface^22^. Methods to enhance the resolution of multimode fibre-based imaging are rapidly being developed^23,24^. However, it is still a great challenge to incorporate these cutting-edge super-resolution technologies into a fibre format.

Here, we propose and experimentally demonstrate fluorescence imaging through a thin MM fibre probe with a spatial resolution beyond the Abbe limit and a temporal resolution beyond the Nyquist limit, meaning that super-resolution and super-speed are achieved at the same time. The proposed approach does not put any specific requirements on the fluorescent labels except the brightness and can be used with any standard dye at a reasonable concentration. Moreover, overcoming the Nyquist limit paves the way towards new designs of flexible super-resolution imaging probes. We exploit the random nature of mode coupling in an MM fibre, the sparsity constraint inherent to any natural image and compressive sensing integrated into a compact fibre probe. The new approach potentially allows minimally invasive deep-tissue imaging with a spatial resolution more than 2 times better than the diffraction limit and an imaging speed 20 times faster than is required by the Nyquist–Shannon limit, opening a new avenue for *in vivo* ultra-fast nanoscale imaging.

## Results

### Super-resolution image reconstruction via compressive sensing

The Abbe diffraction limit^25^ is often considered the theoretical limit of the spatial resolution achievable by a conventional optical system. Nevertheless, it was shown that it is possible to computationally resolve infinitesimally small details^26^. Unfortunately, the existing methods for bandwidth extrapolation^27–29^ are known to be extremely sensitive to noise. As a result, the diffraction limit remains a practical resolution boundary of a simple imaging system^26,30^.

Recently, compressive sensing—a novel imaging paradigm robust to noise in the measured data—has emerged^31,32^. Compressive sensing enables signal acquisition with a large reduction in sampling for signals that have a sparse representation, vastly reducing the number of measurements that need to be done^33–36^. It exploits the fact that many natural signals are sparse on some basis. Compressive sensing can be used to significantly reduce the number of measurements in endo-microscopy applications^37,38^. Remarkably, compressive sensing serves as an effective method for rejecting certain common types of noise, which typically do not have sparse transforms. As a result, super-resolution imaging by using the sparsity constraint is theoretically feasible^39,40^. The idea of super-resolution imaging via compressive sensing was originally demonstrated by using artificial low-pass filtering to measure data^41,42^. Recently, the possibility of reconstructing images in the case where the illumination transverse size is larger than the feature size of the sample was shown, though with a resolution of only tens of micrometres^43,44^.

The spatial resolution of the compressive sensing imaging approach is fundamentally limited by sparsity. In an ideal noise-free oversampling scenario, the resolution improvement can be up to a factor of 1/(2*β*), where *β* is the sparsity parameter (the relative fraction of nonzero basis functions occupied by the original image). The sparsity parameter can be expressed as *β* = *S*/*N*, where *S* is the number of nonzero values and *N* is the total number of basis functions^41,42^. However, it is important to note that the information does not have to be sparse in real space: any mathematical basis that is sufficiently uncorrelated with the sampling basis will work. The majority of natural objects are sparse on some basis. In the following sections, we also consider the practical issues limiting the spatial resolution of the proposed super-resolution endo-microscopy, such as the noise, stability and orthogonality of the sensing basis, and the number of measurements.

### Principles of the optical setup

**Fig. 1:**
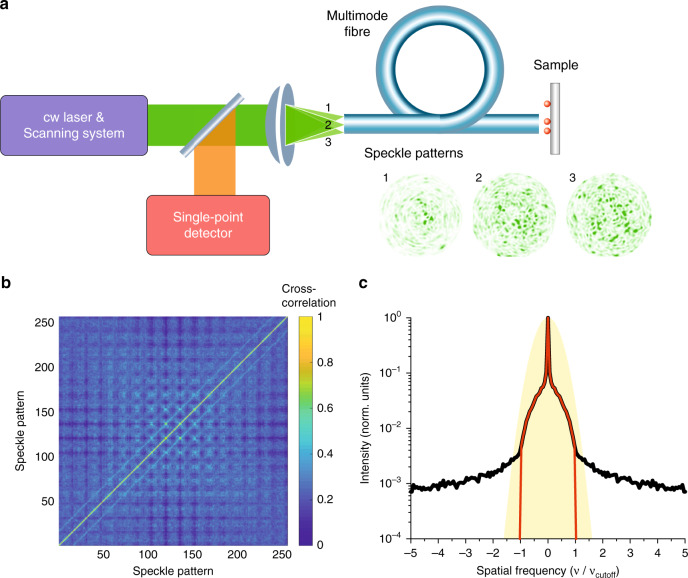
The concept of multimode-fibre-based super-resolution and super-speed endo-microscopy. **a** Experimental setup that consists of three main components: a continuous-wave laser source with a scanning system, an MM fibre probe, and a single-point detector. Pump light is scanned across the fibre input facet, creating different illumination patterns on the fibre output. The total fluorescent response from the sample is collected by the same fibre probe, propagated back, and measured by the single-point detector. **b** Cross-correlation coefficients of different speckle patterns generated in the MM fibre with NA = 0.22 for a total of 256 patterns. **c** The one-dimensional Fourier spectra (*k*_x_ components). Black and red lines represent the average spatial frequency spectra of speckle patterns generated in the MM fibre before and after low-pass filtering, respectively. The filled yellow area shows the spatial power spectrum of an ideal Gaussian beam. The cutoff frequency of the MM fibre ν_cutoff_ = 2NA/*λ*

The idea of MM-fibre-based ultra-fast super-resolution endo-microscopy is sketched in Fig. 1a. The system consists of three main components: a continuous-wave (cw) laser source with a scanning system, an MM fibre probe, and a single-point detector. Pump light is scanned across the fibre input facet, creating different illumination patterns on the fibre output. The total fluorescent response from the sample is collected by the same fibre probe, propagated back and measured by the single-point detector. The “Materials and methods” section and Supplementary Fig. S1 present a complete description of the experimental geometry.

The key component of the proposed approach is a step-index MM fibre. We used two different fibres to demonstrate the independence of the proposed approach to the probe geometry. The first fibre has a silica core of 50 µm in diameter and an NA = 0.22. The Abbe diffraction limit is *λ*/(2NA) = 1.21 μm. The second fibre has a core of 105 µm in diameter, an NA = 0.1, and an Abbe diffraction limit of 2.66 μm. Each MM fibre supports approximately 2000 modes^45^ at a wavelength of 532 nm, leading to a Nyquist limit of approximately 4000 measurements for diffraction-limited sampling.

### Sensing basis provided by mode scrambling in the multimode fibre

One of the fundamental premises underlying compressive imaging is incoherence. Incoherence means that the sampling method should ensure the lowest correlation between any element of the sensing basis and any element of the representation basis. Consequently, to have freedom of choice of the representation basis, we need a universal sensing basis. Random matrices are known to be largely uncorrelated with any fixed basis, which makes them a very good tool for compressive sensing^31^. We create a universal basis for compressive endo-microscopy by using the random nature of mode coupling in an MM fibre^46^. The light field in an MM fibre (step-index circular waveguide) can be expressed as a finite sum of the so-called HE, EH, TE, and TM modes propagating at their own speeds. Different focal positions on the input fibre facet result in different speckle-like mode interferences on the distal facet. An example is presented in Fig. 1a, where three locations of the input beam correspond to three different speckle patterns on the output. A total of 256 patterns are created by scanning a focused input beam over 256 points organized in a square lattice (16 × 16) on the input fibre facet. It is known that the full transmission matrix of a fibre can be measured using a similar scanning procedure^47^, meaning that this experimental geometry potentially addresses all fibre modes. Alternatively, speckle patterns can be generated by any orthogonal bases. A Hadamard basis or a hexagonal scan lattice can be used to achieve a maximum available number of basis vectors.

First, we characterized the orthogonality of the experimentally generated basis by calculating the correlation coefficient between any two measured speckle patterns (see “Materials and methods”). The results for the first fibre are presented in Fig. 1b. The correlation between two random patterns generated in the MM fibre is close to zero, confirming the high level of orthogonality of our speckle basis. However, focal positions on the fibre facet located very close to each other lead to non-negligible correlations between the corresponding speckle patterns. As a result, the experimentally created basis is not ideal. We want to emphasize that in all our simulations, we use these experimentally measured non-ideal speckle-based PSFs and demonstrate that the basis is sufficient for super-resolution endo-microscopy.

Second, we analyzed the spatial frequency content of the speckle patterns produced by the MM fibre with a cutoff frequency ν_cutoff_ = 2NA/*λ*. The spatial power spectra were calculated by a 2D fast Fourier transform (FFT). The average one-dimensional spectrum (*k*_x_ component) is presented in Fig. 1c by the black line. The experimental results are consistent with the theoretical predictions: the measured data at frequencies beyond *ν*_cutoff_ are close to zero and effectively contain only noise. A zoomed-in camera image of the speckle pattern generated by the first MM fibre is presented in Supplementary Fig. S2a. Red arrows indicate the presence of dead pixels on the camera sensor that lead to high-frequency noise. To ensure that we do not introduce any high-frequency components in the simulations of our imaging system, we cut the Fourier spectrum of each measured speckle pattern at *ν*_cutoff_ (the red line in Fig. 1c). Low-pass filtered speckle patterns were recovered by the inverse 2D FFT. A zoomed-in example of the speckle pattern after low-pass filtering is presented in Supplementary Fig. S2b. No more high-frequency components are preserved in the speckle PSF.

Finally, we measured the reproducibility and stability of the speckle patterns while the focused laser beam was switched between different locations on the fibre input. We scanned the focused beam over 100 different locations and recorded 100 different speckle patterns every 3 min. Then, we characterized the reproducibility of the speckle patterns as the cross-correlation coefficient between the pattern measured at time equal to zero and the pattern measured after the time delay and switching between different focal positions. We found that over 45 min, the average cross-correlation coefficient was greater than 0.95, confirming a high level of reproducibility. The results are presented in Supplementary Fig. S4.

### Simulation with low-pass-filtered speckle patterns: beyond the Abbe limit

**Fig. 2:**
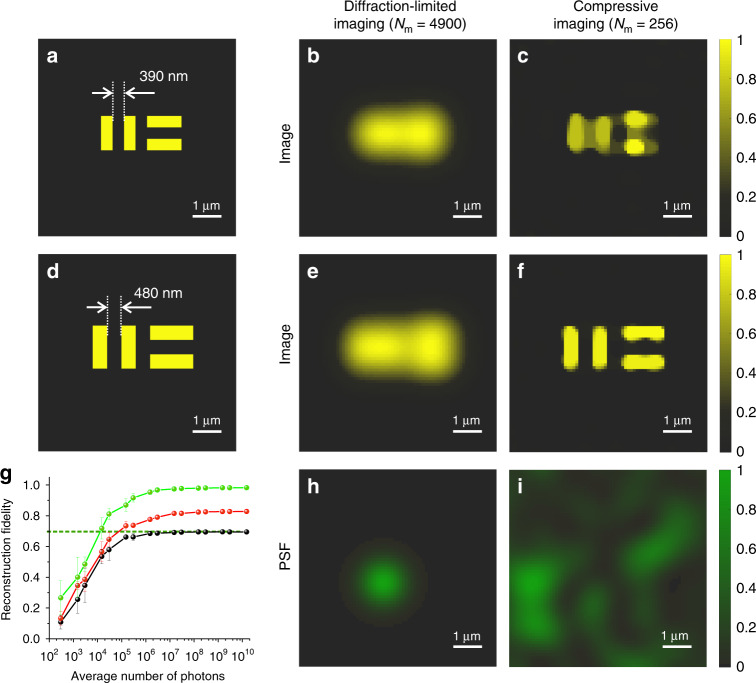
Theoretical comparison of conventional imaging and super-resolution endo-microscopy. **a**, **d** Samples with 390 nm (**a**) and 480 nm (**d**) features. **b**, **e** Conventional diffraction-limited point scan imaging of the sample with 390 nm (**b**) and 490 nm (**e**) features. The total number of measurements *N*_m_ = 4900. **c**, **f** Super-resolution endo-microscopy of the sample with 390 nm (**c**) and 490 nm (**f**) features. The total number of measurements *N*_m_ = 256. **g** The fidelity of compressive endo-microscopy as a function of an average number of “detected” photons in the presence of shot noise for 390 nm (black line), 480 nm (red line), and 580 nm (green line) features. The dashed green line represents the level of the fidelity at which sub-diffraction details could be resolved. **h** Gaussian PSF used for the conventional point scan imaging modality. **i** Example of an experimentally measured and low-pass-filtered speckle-based PSF that was used for super-resolution compressive endo-microscopy. PSFs presented in **h** and **i** have the same cutoff frequency of 2NA/*λ*

As we now demonstrate, exploiting the disorder in MM fibres in combination with the sparsity constraint allows the creation of a compact ultra-fast super-resolution endo-microscope. As a sample, we use a structure consisting of parallel stripes in the horizontal and vertical directions separated by 390 nm (Fig. 2a) and by 480 nm (Fig. 2b). The feature sizes are far below the diffraction limit of the probe we used (*λ*/2NA = 1.21 μm). The field of view (FOV) is 6.8 × 6.8 μm^2^.

First, we simulated conventional point scan imaging using the Gaussian PSF presented in Fig. 2h. The full width at half maximum (FWHM) of the Gaussian PSF corresponds to the diffraction limit of the MM fibre. The spatial power spectrum is represented in Fig. 1c by the filled yellow area. We calculated the overlap integral between the Gaussian PSF and the sample for different positions of the pump beam. A total of 4900 values were calculated for each sample. The resulting images are presented in Fig. 2b, e for 390 and 480 nm feature sizes, respectively. Conventional microscopy does not resolve any sub-diffraction details.

Second, we simulated super-resolution compressive imaging through an MM fibre. As a sensing basis, we used the experimentally measured and low-pass-filtered speckle patterns. An example is presented in Fig. 2i. We calculated the response for each speckle pattern of our sensing basis. A total of only 256 values were calculated for each sample. The collected data were scaled in such a way as to represent the number of “detected” photons. We added shot noise by generating random numbers from the Poisson distribution specified by the number of photons. First, we analyzed the average number of photons, i.e., 10^10^. To retrieve a 4900 pixel image from only 256 measurements, we used the reconstruction procedure described in the “Materials and methods” section. The acquired images are presented in Fig. 2c, f for 390 and 480 nm feature sizes, respectively. In contrast to conventional imaging, all sub-diffraction details are resolved even if the distance between them is more than 3 times smaller than the diffraction limit. The spatial power spectra of the acquired images resemble those of the original sample and spread way beyond the cutoff frequency of our optical system (*ν*_cutoff_); see Supplementary Fig. S3.

### Robustness to noise

We analyzed the quality of super-resolution compressive endo-microscopy for different noise levels. We used three samples with sub-diffraction features separated by 390 nm (see Fig. 2a), 480 nm (see Fig. 2d), and 580 nm. We simulated the super-resolution compressive endo-microscopy by using different average numbers of “detected” photons per measurement, ranging from 10^2^ to 10^10^ (leading to a different signal-to-noise ratio according to the Poisson statistics). Supplementary Movie 1 demonstrates how the acquired image improves with an increase in the number of detected photons for the 480 nm feature size. For hundreds of photons, the image contains only noise; however, at approximately 10^5^ photons, all four stripes start to be resolved. Finally, from approximately 10^8^ photons, we obtain a good image presented in Fig. 2f.

For each acquired image, we retrieved the reconstruction fidelity by calculating the cross-correlation coefficient, *k*_s_, between the recorded image and the original sample (see “Materials and methods”). The fidelities as a function of the number of photons for the 390, 480, and 580 nm features are presented in Fig. 2g in black, red, and green, respectively. The dashed line in Fig. 2g represents the level at which we can observe sub-diffraction details. The graph shows a similar behaviour for different samples, with the main distinction being in the converging quality: the 580 nm features can be imaged with high fidelity, whereas the 390 nm features can barely be resolved.

### Simulation with low-pass-filtered speckle patterns: beyond the Nyquist limit

To analyze the super-speed property of our super-resolution endo-microscopy, we address the following questions: how many measurements are actually required to resolve the sub-diffraction features of our sample and how are the imaging speed and the resolution related to each other? In our simulations, we kept the general structure of interest the same (see Fig. 2d) and increased the FOV and the sparsity by adding zero pixels. The total length of each dimension of the FOV varied from 6.4 to 10.6 μm, and the total number of pixels *N*_p_ varied from 4356 to 12,100. For each sample size, we simulated the super-resolution approach to endo-microscopy for total numbers of experimentally measured speckle patterns, *N*_m_, from 50 to 256. We performed simulations for an average number of “detected” photons of 10^10^. It is important to mention that we always kept the number of measurements far below the Nyquist limit, with a minimum of 0.4% to a maximum of 6% of the information, and the sample contained features 2.5 times smaller than the diffraction limit of the fibre.

**Fig. 3:**
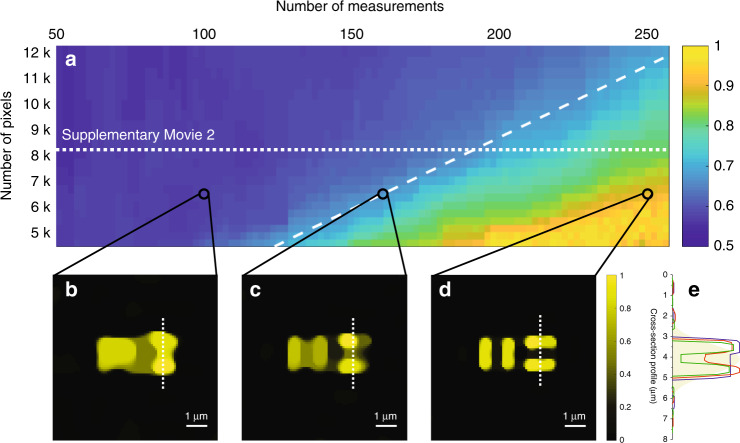
Theoretical analysis of the trade-off between the imaging speed and resolution below the diffraction limit. **a** Fidelity of the super-resolution compressive endo-microscopy as a function of the total number of pixels in the FOV (*N*_p_) and the total number of measurements (*N*_m_) for a sample with a feature size of 480 nm. The white dashed line shows that the *N*_m_ needed for sub-diffraction imaging is linearly proportional to *N*_p_. The recorded images of the sample for *N*_p_ = 8100 (white dotted line) are presented in Supplementary Movie 2 as a function of the number of measurements. **b**–**d** Results of the compressive endo-microscopy simulated by using experimentally measured and low-pass-filtered speckle patterns on the MM fibre output and in the presence of shot noise for the sample with *N*_p_ = 6400: **b** *N*_m_ = 100; **c** *N*_m_ = 160; and **d** *N*_m_ = 250. **e** Cross-sectional plots along the dashed lines in **b**, **c**, and **d** are represented by the blue, red, and green lines, respectively. The filled yellow area represents a cross-section of a conventional diffraction-limited image

For each image, we characterized the reconstruction fidelity by the cross-correlation coefficient, *k*_s_, between the acquired image and the original sample. Figure 3a shows the image quality as a function of the total number of pixels *N*_p_ and the total number of measurements *N*_m_. Low values of the cross-correlation coefficient (blue) represent images with a diffraction-limited resolution. An example is presented in Fig. 3b (*N*_m_ = 100 and *N*_p_ = 6400). A cross-correlation coefficient of more than approximately 0.7 (light blue) corresponds to an image that still resolves the sub-diffraction features of our object. An example is presented in Fig. 3c (*N*_m_ = 160 and *N*_p_ = 6400). Yellow (*k*_s_ close to 1) represents a very good reconstruction: an example for *N*_m_ = 250 and *N*_p_ = 6400 is presented in Fig. 3d. The total number of measurements *N*_m_ that is needed for sub-diffraction imaging is linearly proportional to the total number of pixels *N*_p_ (white dashed line), while *N*_m_ ≪ *N*_p_. The cross-sections over two stripes in the images in Fig. 3b–d are presented in Fig. 3e by the blue, red, and green lines, respectively. The cross-section of the image for standard diffraction-limited microscopy is presented in Fig. 3e by the filled yellow area. The recorded images of the sample with a 480 nm feature size as a function of the number of measurements for *N*_p_ = 8100 are presented in Supplementary Movie 2. We can see the improvement in the image quality as the number of measurements increased.

### Experimental demonstration of endo-microscopy beyond the Abbe and Nyquist limits

**Fig. 4:**
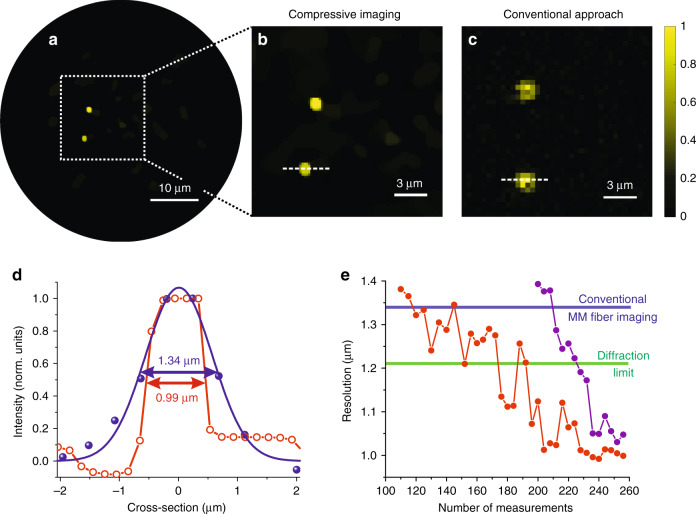
Super-resolution and super-speed compressive endo-microscopy through the MM fibre with NA = 0.22. **a**–**c** Images of two 450-nm-diameter fluorescent spheres through the MM fibre probe obtained by **a**, **b** the proposed super-resolution endo-microscopy (full FOV is presented in **a** and zoomed-in area of interest is presented in **b**) and **c** the state-of-the-art raster scan wavefront shaping-based endo-microscopy. **d** Cross-sectional plots along the dashed lines in (**b** open red circles) and (**c** filled blue circles). The blue line is a Gaussian fit to the data, and the red line interpolates the data. **e** Resolution of compressive endo-microscopy as a function of the total number of measurements for two fluorescent spheres is presented by different colours. The diffraction limit is represented by the solid green line, and the resolution achieved in the same setup by the state-of-the-art endo-microscopy method is represented by the solid blue line

In the first set of measurements, we demonstrated super-resolution and super-speed compressive endo-microscopy through the first MM fibre with NA = 0.22. The details of the imaging procedure are given in the “Materials and methods” section. Similar to our simulations, we performed only 256 measurements and reconstructed the full FOV, consisting of more than 4000 pixels. The result is presented in Fig. 4a, and a zoomed-in area of interest is presented in Fig. 4b. We compared the results of the proposed approach with those of the state-of-the-art method of MM fibre imaging based on complex wavefront shaping^6^, where the image is retrieved after point-by-point scanning of a focused laser beam along the MM fibre output facet. For wavefront shaping-based endo-microscopy, we used the same setup and the same fibre probe to retain the same numerical aperture and the resolution of the system. We did not use the same sample because the small spheres demonstrated pronounced photobleaching of the fluorescent signal that may influence the performance of the second measurement. Instead, we used a similar sample consisting of spheres with exactly the same diameter of 450 nm (standard deviation was 9 nm). The result (an enlarged view of the area of interest) is presented in Fig. 4c.

A cross-section of a fluorescent sphere imaged with the conventional laser scanning endo-microscopy approach is represented in Fig. 4d by blue circles. It follows a Gaussian shape (blue solid line) with an FWHM = 1.34 μm, which is slightly above the diffraction limit due to inaccuracies in the wavefront shaping and the distance between the fibre facet and the sample. The cross-section of a fluorescent sphere imaged with compressive super-resolution endo-microscopy is represented in Fig. 4d by open red circles. In contrast to the state-of-the-art method, it has very sharp edges and does not follow the conventional Gaussian shape. The FWHM of 0.99 μm was estimated using interpolation. The measured sphere diameter was 20% below the diffraction limit of our optical system. Simultaneously, we used only approximately 5% of the information, meaning the imaging speed was 20 times faster than would be needed for scanning-based imaging with any other technique.

In the second set of measurements, we experimentally analyzed the intrinsic trade-off between the spatial resolution and the imaging speed. We repeated the compressive endo-microscopy experiment for a different number of measurements varying from 110 to 256. The results are presented in Fig. 4e, where the experimentally measured resolution of the proposed approach for two fluorescent spheres is plotted as a function of the number of measurements. The diffraction limit is presented by the solid green line, and the resolution achieved under the same setup by the state-of-the-art endo-microscopy method is presented by the solid blue line. We see that by reducing the number of measurements and keeping the same sample, we reduce the resolution, as was predicted by our simulations.

We note that the number of pixels in the images where the results of the reported and conventional methods are compared (Fig. 4b, c) are not identical. Differences in the pixel number are a direct consequence of differences in the imaging techniques. For each method, the pixel size was chosen as a compromise between the imaging (or reconstruction) speed and the resolution. Here and later, we ensured that the pixel sizes were always several times smaller than the best achievable spatial resolution.

**Fig. 5:**
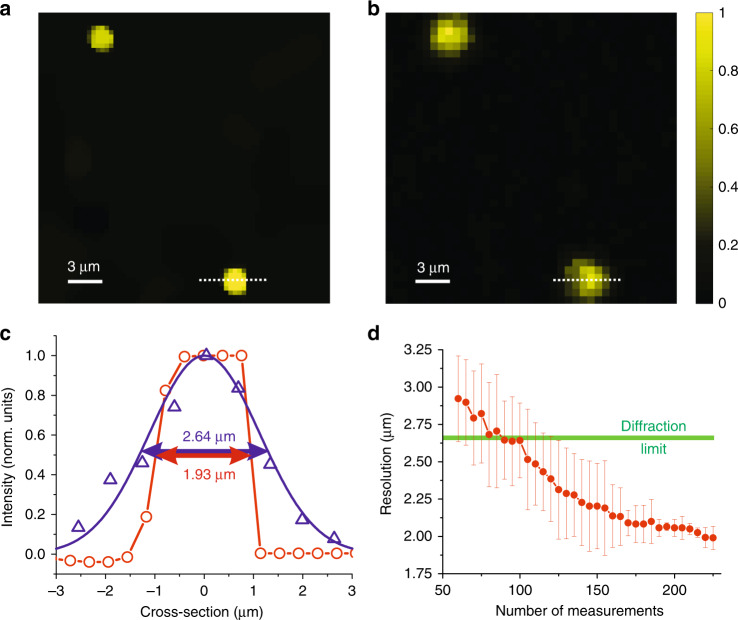
Super-resolution and super-speed compressive endo-microscopy through the MM fibre with NA = 0.1. **a**, **b** Images of two fluorescent spheres through the MM fibre probe obtained by **a** the proposed super-resolution compressive endo-microscopy and **b** the state-of-the-art raster scan wavefront shaping-based endo-microscopy. **c** Cross-sectional plots along the dashed lines in (**a** open red circles) and (**b** open blue triangles). The blue line is a Gaussian fit to the data, and the red line interpolates the data. **d** Resolution of compressive endo-microscopy as a function of the total number of measurements averaged over four fluorescent spheres. The error bars represent the standard deviation. The diffraction limit is represented by the solid green line

Equivalent measurements were taken for an MM fibre with a core of 105 µm and NA = 0.1. This fibre has a diffraction limit of 2.66 µm and supports approximately the same total number of modes as the first one. Consequently, despite the 2-fold larger FOV, we expect a similar resolution improvement according to our simulations. To demonstrate this, we compare the performance of the proposed approach (Fig. 5a) with that of the state-of-the-art endo-microscopy (Fig. 5b). We used the same setup and the same sample for both measurements.

Cross-sectional plots along the dashed lines in Fig. 5a, b are represented in Fig. 5c by blue triangles and red circles, respectively. The FWHM for the proposed endo-microscopy approach is 1.93 μm, which is approximately 25% below the diffraction limit of our optical system. Simultaneously, we performed only 225 measurements, meaning the imaging speed was 20 times faster than would be needed for scanning-based imaging. The trade-off between the spatial resolution and the imaging speed is presented in Fig. 5d, where the experimentally measured resolution averaged over four fluorescent spheres is plotted as a function of the total number of measurements. The error bars represent the standard deviation. The diffraction limit is represented by the solid green line.

### Improving the resolution by reducing the FOV and the noise level

Our theoretical analysis shows that the resolution depends on the total number of measurements and can be significantly improved by reducing the total FOV for the same number of speckle patterns forming the basis. In the final set of measurements, we experimentally demonstrated this effect by restricting the image reconstruction to a smaller area of the fibre. We used a fibre with NA = 0.1 (diffraction limit of 2.66 μm) and a core diameter of 105 μm, but we reconstructed the image in only a 30 × 30 micron area on the fibre facet, while we made sure that no fluorescent spheres were present outside the reconstructed area. In this manner, we artificially mimicked a fibre with a small FOV. We used larger fluorescent spheres (approximately 1.5 μm in diameter) to reduce the noise level.

**Fig. 6:**
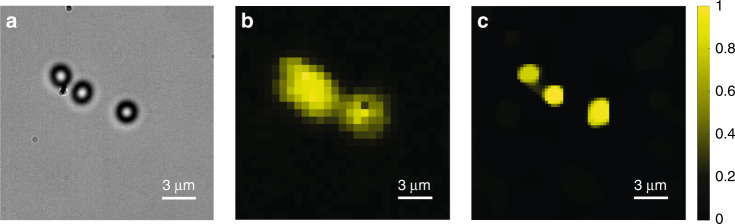
Resolution improvement of compressive endo-microscopy by reducing the FOV and the noise level. **a** Bright-field image of the fluorescent spheres. The separations between the left and middle spheres and between the middle and right spheres are approximately 1 μm and less than 3 μm, respectively. The diffraction limit of the system is 2.66 μm. **b**, **c** Images obtained through an MM fibre probe in the endoscopic regime by **b** state-of-the-art raster scan wavefront shaping-based endo-microscopy and **c** the proposed super-resolution compressive endo-microscopy with a reduced FOV

The bright-field image of the sample is presented in Fig. 6a. The separation between the left and middle spheres was approximately 1 μm, and the separation between the middle and right spheres was less than 3 μm. We repeated the experimental procedures described in the previous section. The image of the sample obtained with the state-of-the-art endo-microscopy based on wavefront shaping is presented in Fig. 6b (zoomed-in area of interest). We see that the left and middle spheres are completely unresolvable and that the middle and right spheres are hardly resolved, as expected. We used the same fibre probe and the same sample for the super-resolution endo-microscopy experiments. The result (the enlarged area of interest) is presented in Fig. 6c. All three spheres were fully resolved.

## Discussion

We have demonstrated that the compressive sensing approach in combination with an MM fibre probe can provide imaging beyond the Abbe and Nyquist limits simultaneously in an endoscopic configuration. Our simulations show that the spatial resolution can be improved by more than 3 times below the diffraction limit and simultaneously that the speed can be made more than 20 times faster than that dictated by the Nyquist limit. Supplementary Movie 3 presents experimentally acquired images as a function of the total number of measurements for super-resolution compressive endo-microscopy (left) and for the state-of-the-art point scan endo-microscopy based on wavefront shaping (right). The experiments were performed under the same setup, same signal level and same fibre probe. This movie clearly demonstrates the advantages of super-resolution compressive endo-microscopy: significantly fewer measurements are needed to obtain a full FOV image with a resolution below the diffraction limit. In the case of the conventional approach, the same number of measurements allows visualization of only a small fraction of the FOV with a diffraction-limited resolution at best.

The best possible spatial resolution of the compressive sensing approach is fundamentally limited by the theoretical resolution enhancement factor, 1/(2*β*), as was shown by Gazit et al.^41^. In our simulations and experiments, *β* was estimated to be 0.06 or smaller, meaning that the theoretical limit of the resolution enhancement factor was 8 or more. However, this limit was derived for an ideal noise-free oversampling scenario. We incorporated practical limitations of the resolution improvements, such as the noise, stability, and reproducibility of the basis vectors, orthogonality of the sampling basis, and imaging speed (number of measurements). We concluded (see Figs. 3, 4e, and 5d) that the imaging speed and the resolution are closely related: the better the resolution we want to obtain, the larger the number of measurements we need to perform. We carefully analyzed the effect of the shot noise (see Fig. 2g and Supplementary Movie 1). The estimation of the fundamental spatial resolution limit as a function of all these parameters is a subject of further investigation. It is important to note that we demonstrated that the spatial resolution of the proposed compressive endo-microscopy is not limited to a 2-fold improvement.

We highlight that overcoming the Nyquist limit not only helps to improve the imaging speed but also opens up new avenues for the development of flexible ultra-thin super-resolution imaging probes. By overcoming the Nyquist limit, we significantly decrease the necessary number of input modes that provide super-resolution imaging through an MM fibre. Our results show that only approximately 250 speckle patterns are needed to image a 50 μm diameter area with a resolution below the diffraction limit. This number is more than an order of magnitude smaller than the number of complex wavefronts that are needed for the state-of-the-art diffraction-limited endo-microscopy-based holographic control of light propagation^12^. To beat the deleterious effects of MM fibre bending, we need to organize a stable delivery of only 250 different single modes (and not complex wavefronts) to the entry facet of the MM fibre. This process can be carried out in many ways. As an example, a short rigid MM fibre and a long bundle of single-mode fibres that are immune to bending can be fused together, where the excitation light is scanned over the entry facet of the fibre bundle. This will generate stable speckle patterns at the exit facet of the rigid MM fibre. For a fibre probe of 50 μm in diameter and 250 input modes, a fibre bundle with a 3 μm distance between cores provides a stable light delivery suitable for super-resolution endo-microscopy. These fibre bundles are commercially available. Implementing a fibre bundle instead of point-by-point scanning through a microscope objective conceptually does not change the approach.

Remarkably, the new super-resolution endo-microscopy approach does not use any specific properties of the fluorescent label typical for many super-resolution techniques such as depletion (STED) or stochastic activation of the molecular fluorescent (PALM). The main limiting factor related to the sample is the number of registered photons: the sample should be bright enough to provide more than 10^4^ multiplied by *N*_m_ photons. The method in its present form is not suitable for single-molecule imaging because a conventional dye molecule usually emits only 10^6^ photons before being bleached. Super-resolution endo-microscopy is ready to be implemented in a variety of fluorescent and label-free modalities of microscopy.

## Materials and methods

### Experimental setup

All experiments were performed using standard step-index MM fibres [Thorlabs, FG050UGA and FG105LVA] with a silica core diameter of 50 µm, NA = 0.22, and of 105 µm, NA = 0.1. The fibres were not straight, and a slight curve was introduced to improve cross-mode coupling; the length of each fibre was approximately 20 cm. A detailed sketch of the experimental setup is presented in Supplementary Fig. S1. As a pump, we used the continuous-wave linearly polarized second harmonic output of a Nd:YAG laser [Cobolt Samba] with a wavelength of 532 nm and power below 1 mW. As a laser scanning element for endo-microscopy, we used a spatial light modulator: 1920 × 1200 Vialux V4100 digital micromirror device (DMD). This choice stems from the ability of the DMD to perform phase control, which is required for the comparison of our new approach with the state-of-the-art wavefront-shaping-based method of high-resolution endo-microscopy. The beam was expanded by the telescope to match the area of the DMD. Two lenses were placed in a 4*f* configuration to image the DMD on the back focal plane of a 20× (NA = 0.4) or 10× (NA = 0.25) objective that coupled the light into the MM fibre. A pinhole in the Fourier plane blocked all but the 1st diffraction order, encoding the desired phase or amplitude distribution. The DMD was used to control the spatial phase (for the state-of-the-art endo-microscopy) or the spatial amplitude (for the super-resolution compressive endo-microscopy) of a beam on the input facet of the fibre. A 50× (NA = 0.75) or 40× (NA = 0.6) objective was used to collect light from the fibre output (transmission side). The camera imaged the output facet of the MM and was used for pre-calibration. The same MM fibre was used to collect the total fluorescent response and propagate it in the backward direction to the detection system. We used a dichroic mirror to separate the fluorescent signal and an additional Notch filter to remove the pump light. For detection, a standard avalanche photodiode [Thorlabs, APD410A2] was used in combination with a low-noise preamplifier [Stanford Research].

As a sample, we used fluorescent microparticles (PS-FluoRed-Fi144–2) with a diameter of 450 ± 9 nm and fluorescent microparticles (Nile red) with a diameter of 1.1–1.5 μm. Microparticles were randomly distributed over a glass substrate.

### State-of-the-art wavefront-shaping-based endo-microscopy

During the pre-calibration step, the complex wavefront shaping algorithm^7^ was used to create tightly focused spots on the fibre output. The phase masks corresponding to focal spots at different positions on the output fibre facet were calculated and stored. After the wavefront shaping procedure, the sample was placed as close to the fibre facet as possible without touching. The sample image was acquired in the endoscopy configuration by sequentially applying the recorded phase masks to the DMD and detecting the total fluorescent signal. As a result, the image of the sample was reconstructed pixel-by-pixel, and approximately 4000 measurements were required to provide an image with diffraction-limited resolution over the full FOV, according to the Nyquist–Shannon sampling theorem.

### Compressive endo-microscopy

The pre-calibration procedure consisted of recording a set of 256 speckle patterns on the output facet of the MM fibre for different positions of the focal spot on the fibre input facet, organized in a 16 × 16 square lattice. The background signal that corresponded to each speckle pattern was also recorded. After the pre-calibration, the sample was placed as close to the output fibre facet as possible without touching. Super-resolution and super-speed compressive endo-microscopy was performed by scanning the focal spot at the input fibre facet in the same way as during the pre-calibration procedure. The total fluorescent response for each speckle pattern collected by the MM fibre probe was measured by a single-pixel detector. A total of 60–256 measurements were taken in different experiments. To retrieve an image, we used the reconstruction procedure described in the following section.

### Image reconstruction

The fundamental problem is to recover a vector $x \in {\Bbb R}^n$ from data *y: y* = *Ax* + *z*, where *A* is a known *m* × *n* “sensing matrix”, which is used in the measurements, *z* is an unknown error term, *n* is the number of pixels, and *m* is the number of “measurements”. We are interested in the case where *m* ≪ *n*, and the distance between two neighbouring pixels Δ*n* is smaller than the Abbe diffraction limit *λ*/2NA, where *λ* is the wavelength and NA is the numerical aperture of our optical system.

We used an established procedure as the solution to the *l*_1_ minimization problem^48^. Among all objects consistent with the data, we chose the one whose coefficient sequence had a minimal *l*_1_ norm:

1 $${\mathop{\rm{min}}\limits_{\tilde{x} \in {\Bbb R }^{n}}} \Vert{\tilde{x}}\Vert_{l_{1}}\;{\mathrm{subject}}\;{\mathrm{to}}\; A{\tilde{x}} = y$$

This is a standard linear programming problem that can be solved in polynomial time using one of the many existing software packages. We used the open software algorithm “*l*_1_ magic” from Stanford.edu^49^.

### Cross-correlation coefficient

We calculated the correlation coefficient *r* between any two measured speckle patterns by using the following equation:

2 $$r = \frac{{{\sum} {\left( {a - \overline a } \right)} \left( {b - \overline b } \right)}}{{\sqrt {{\sum} {\left( {a - \overline a } \right)^2} {\sum} {\left( {b - \overline b } \right)^2} } }}$$

where *a* and *b* are images of speckles on the output facet of an MM fibre and $\bar a$ and $\bar b$ are their means. A total of 256 speckle patterns were created by scanning a focused input beam over 256 points organized in a square lattice (16 × 16) on the input fibre facet. Correlation coefficients were calculated for all pairs of speckle patterns, and the results are presented in Fig. 1b.

We also used the cross-correlation coefficient to characterize the fidelity of the acquired image. The reconstruction fidelity was calculated as the cross-correlation coefficient between the recorded image and the original sample. In that case, *a* and *b* were the recorded image and the original sample, respectively, and $\bar a$ and $\bar b$ were their means.

## Supplementary information

Supplemental material

Supplementary Movie 1

Supplementary Movie 2

Supplementary Movie 3

## Data Availability

The data that support the findings of this study are available from the corresponding author upon reasonable request.
