# Supplementary material for: Endo-microscopy beyond the Abbe and Nyquist limits
Source: Light Sci Appl. 2020 May 7;9:81. doi: 10.1038/s41377-020-0308-x (PMC7206071; doi:10.1038/s41377-020-0308-x)
Supplement: Supplementary file 1 — Supplemental material [file 41377_2020_308_MOESM1_ESM.docx]

**Endo-microscopy beyond the Abbe and Nyquist limits: supplementary material**

Lyubov V. Amitonova^1,2,*^ and Johannes F. de Boer^1^

^1^*LaserLaB, Department of Physics and Astronomy, Vrije Universiteit Amsterdam, De Boelelaan 1081, 1081 HV Amsterdam, The Netherlands*

^2^*Advanced Research Center for Nanolithography (ARCNL), Science Park 106, 1098 XG, Amsterdam, The Netherlands*

^*^e-mail: [l.amitonova@vu.nl](mailto:l.amitonova@vu.nl)

**Fig. S1 | Experimental setup.** The experimental setup consists of 3 main components: a continuous-wave (cw) laser source with a scanning system, a MM fibre probe, and a single-point detector. Pump light is scanned across the fibre input facet creating different illumination patterns on the fibre output. The super-resolution and super-speed compressive endo-microscopy is performed by using the scanning system (DMD) only for amplitude control. The state-of-the-art wavefront shaping based point scan endo-microscopy is performed by using the scanning system (DMD) for spatial phase control only. The total fluorescent response from the sample is collected by the same fibre probe, propagated back and measured by the single-point detector. Bright-field microscopy is used for reference. The CCD camera is used for pre-calibration. DMD, digital micromirror device; M, mirrors; P, pinhole; DM, dichroic mirror; Obj, objectives; APD, avalanche photodiode.





**Fig. S2 | Analysis of the measured speckle patterns.** Zoomed-in camera image of the speckle pattern generated by the MM fibre **a,** before and **b,** after low-pass filtering by cutting the spatial power spectrum at ν_cutoff_ = 2NA/λ. Red arrows indicate the presence of dead pixels on the camera sensor that lead to high-frequency noise. After low-pass filtering, there are no high-frequency components present in the speckle PSF.

**Fig. S3 | Spatial power spectra cross-sections. a,** Cross-section of the spatial power spectra of the sample with a feature size of 390 nm. **b,** Cross-section of the spatial power spectra of the sample with a feature size of 480 nm. Red lines represent the average low-pass filtered spatial power spectra of the speckle patterns generated in the MM fibre and used for imaging. Green lines represent spatial power spectra of the original samples. Violet lines show the spatial power spectra of the image acquired by super-resolution compressive endo-microscopy. The cutoff frequency is equal to 2NA/λ.

**Fig. S4 | Reproducibility and stability of speckle patterns.** **a**, Cross-correlation coefficient between the speckle pattern measured at zero time and the speckle pattern measured after sequentially switching to 99 other positions, averaged over 100 positions of the focal spot on the fibre input facet. **b-e** Examples of the recorded speckle patterns for a particular position of the focal spot on the fibre input facet for 0 (b), 12 (c), 24 (d) and 45 (e) minutes.
